# Decoding Potential Cuproptosis-Related Genes in Sarcopenia: A Multi-Omics Network Analysis

**DOI:** 10.3390/biology14121642

**Published:** 2025-11-21

**Authors:** Hongyu Yan, Long Shi, Yang Li, Zhiwen Zhang

**Affiliations:** 1 College of Acupuncture and Orthopedics, Hubei University of Chinese Medicine, Wuhan 430061, China; 2332703537@stmail.hbucm.edu.cn (H.Y.); monooxx@hotmail.com (L.S.); 2 Hubei Shizhen Laboratory, Wuhan 430061, China; dyy3327@163.com; 3 Affiliated Hospital of Hubei University of Chinese Medicine, Hubei Provincial Hospital of Traditional Chinese Medicine, Wuhan 430061, China; 4 Hubei Key Laboratory of Theory and Application Research of Liver and Kidney in Traditional Chinese Medicine, Wuhan 430061, China

**Keywords:** sarcopenia, cuproptosis, *SLC25A12*, *PABPC4*, machine learning

## Abstract

Sarcopenia is a common age-related skeletal muscle disorder that lacks diagnostic and therapeutic options. Emerging evidence suggests that cuproptosis, a copper-dependent form of regulated cell death, contributes to muscle atrophy, yet the underlying associations remain poorly understood. To address this gap, we integrated two GEO datasets (GSE1428 and GSE25941) for differential expression analysis and applied weighted gene co-expression network analysis (WGCNA) to identify disease-related modules. Cuproptosis-related genes (CRGs) from GeneCards database were intersected with DEGs and WGCNA gene modules to obtain sarcopenia-associated cuproptosis DEGs (SAR-CUP DEGs). Functional enrichment was performed using GO, KEGG, GSEA and GSVA. Hub genes were further identified through three machine learning algorithms (LASSO, RF, and SVM). Regulatory networks were constructed via NetworkAnalyst and GeneMANIA database. A diagnostic model was also developed and later validated in an independent dataset (GSE136344). Experimental validation was performed in a D-galactose-induced sarcopenia cell model. We identified 367 DEGs and 7 co-expression modules, among which 14 SAR-CUP DEGs were mainly enriched in mitochondrial energy metabolism pathways. Machine learning methods highlighted *SLC25A12* and *PABPC4* as hub genes. Regulatory network analysis revealed key modulators, such as FOXC1, miR-16-5p, GOT2, and GOT1. Diagnostic performance analysis demonstrated strong predictive value for *SLC25A12* (AUC = 0.879) and *PABPC4* (AUC = 0.858), and RT-qPCR confirmed their downregulation in the sarcopenia cell model (*p* < 0.01). In conclusion, *SLC25A12* and *PABPC4* are promising biomarkers linking copper metabolism dysregulation with sarcopenia, offering potential targets for diagnosis and therapy.

## Simple Summary

Sarcopenia is an age-related muscle wasting condition that currently lacks specific diagnostic biomarkers and effective treatments. This study investigated a new idea: whether a specific type of cell death linked to copper, called cuproptosis, plays a role in causing this condition. Our goal was to find key genes that connect copper metabolism to sarcopenia. By analyzing public genetic data and using advanced bioinformatics methods, we identified two genes, *SLC25A12* and *PABPC4*, as central players. These genes appear to be involved in the mitochondrial energy production problems seen in the disease. We confirmed that both genes are significantly down-regulated in sarcopenia muscle cells. A diagnostic model based on these genes showed high accuracy in identifying the disease. These findings provide new understanding of sarcopenia and open the door for strategies that might involve monitoring copper levels and developing new drugs or nutritional plans to help prevent and treat sarcopenia in the elderly.

## 1. Introduction

Sarcopenia is a progressive skeletal muscle disease featuring accelerated declines in muscle mass and function [1]. Aging is a major contributor to this condition [2]. Among the elderly, the prevalence of sarcopenia ranges from 5% to 17%, rising significantly in institutionalized older populations [2,3]. Patients with sarcopenia face not only reduced mobility and impaired quality of life, but also an increased risk of comorbidities such as diabetes [4], osteoporosis [5], and Alzheimer’s disease [6], leading to a substantial increase in all-cause mortality. Growing interest in sarcopenia has emerged since its official assignment of an international classification of diseases-10 clinical modification (ICD10-CM) code in 2016 [7]. However, due to the complex and diverse pathogenesis of sarcopenia and the current inconsistency in diagnostic criteria [8,9,10], no specific diagnostic biomarkers are currently available. The diagnosis of sarcopenia relies heavily on muscle mass and strength as measured by metrics such as bioelectrical impedance (BIA) or dual-energy x-ray absorptiometry (DXA), which are highly conditional on the researcher’s available resources [11]. Consequently, no medications have proven effective for preventing or treating sarcopenia [12]. This lack of consensus not only leads to inconsistencies in clinical decision-making, but also complicates the development of standardized treatment protocols and challenges the comparison and evaluation of therapeutic efficacy [13]. Therefore, a thorough comprehension of the molecular mechanisms underlying sarcopenia and the exploration of specific biomarkers have become urgent priorities in current research.

Copper ions (Cu^2+^) are essential for skeletal muscle physiology as they regulate myoblast proliferation and differentiation and support metabolic homeostasis in mature muscle cells through their roles in various enzymes [14,15]. However, excessive accumulation of intracellular copper ions may trigger cuproptosis, a new form of regulated cell death first introduced in 2022 [16]. In contrast to other well-known cell death mechanisms such as apoptosis, necroptosis, and ferroptosis, cuproptosis is distinguished by its reliance on Cu^2+^ buildup, close association with mitochondrial function, the involvement of lipoylated protein aggregation, and oxidative damage [17]. Notably, mitochondrial dysfunction, oxidative stress, chronic inflammation, and disturbed protein metabolism are recognized as important pathological processes in sarcopenia [18,19,20,21]. This suggests that cuproptosis may be important in the pathogenesis of sarcopenia. Aging cells typically exhibit dysregulated expression of copper transporters, leading to increased copper influx and diminished efflux, which ultimately drives intracellular copper accumulation [22]. In skeletal muscle, this accumulation can be aggravated by the tissue’s high copper storage capacity [23]. Evidence has demonstrated that the elevated serum copper levels have been linked to impaired muscle health [24,25], which may lead to an increase in intracellular copper ions. Therefore, abnormal copper metabolism may contribute to muscle wasting through cuproptosis pathways. Nevertheless, the mechanistic relationship between cuproptosis and sarcopenia remains incompletely understood, representing a significant knowledge gap in current research.

This study aimed to elucidate the potential role of cuproptosis in sarcopenia through an integrative analytical approach. We systematically analyzed sarcopenia-related transcriptomic datasets from the gene expression omnibus (GEO) database and identified key cuproptosis-related biomarkers using machine learning algorithms. To further investigate their functional relevance, we explored the regulatory mechanisms of these genes and constructed a diagnostic model, which was subsequently validated by an independent dataset. In addition, a D-gal-induced sarcopenia cell model confirmed the differential expression of the hub genes, supporting a mechanistic link between cuproptosis and muscle degeneration. Collectively, these findings not only enhance our understanding of the molecular basis of sarcopenia but also highlight potential candidate biomarkers for its diagnosis and clinical management.

## 2. Materials and Methods

### 2.1. Data Collection and Preprocessing

Microarray datasets related to sarcopenia were obtained from the GEO database (http://www.ncbi.nlm.nih.gov/geo/, accessed on 8 July 2025) under the accession number of GSE1428 [26], GSE25941 [27], and GSE136344 [28]. The search query was as follows: (“sarcopenia” [All Fields] OR “aging muscle” [All Fields] OR “muscle transcriptome” [All Fields]) AND “vastus lateralis muscle” [All Fields] AND “Homo sapiens” [porgn] AND “Expression profiling by array” [Filter]. All included datasets were screened and cross-verified by two independent researchers. Table 1 details the characteristics of the selected datasets.

The datasets underwent cleaning and normalization procedures prior to analysis. GSE1428 and GSE25941 were merged via the R package “inSilicoMerging” [29] (version 1.2.0) and the batch effects were adjusted using the Combat method [30] via the R package “sva” (version 3.50.0). The merged dataset was served as the training set, comprising 25 control samples and 33 sarcopenia samples. Selected samples from GSE136344 dataset were chosen as the validation set, which included 11 control samples and 12 sarcopenia samples. A total of 2538 cuproptosis-associated protein coding genes were initially identified from the GeneCards database (https://www.genecards.org/, accessed on 8 July 2025) using the keywords “copper death” and “cuproptosis”, with 2504 unique genes retained after duplicate removal.

### 2.2. Differential Expression Analysis

Differentially expressed genes (DEGs) in sarcopenia were identified using the “limma” package [31] (version 3.52.2), by comparing transcriptional profiles between the control and sarcopenia groups in the training set. The screening criteria were as follows: |log_2_ fold change (FC)| > 0.5 and adjusted *p*-value (*p*.adj) < 0.05. The volcano plot and heatmap of the DEGs were generated by the “ggplot2” package [32] (version 3.4.4) and “ComplexHeatmap” package [33] (version 2.13.1), respectively.

### 2.3. Gene Set Enrichment Analysis (GSEA)

To explore the biological functions and pathways between two groups, we performed GSEA [34] via the “clusterProfiler” package [35] (version 4.10.0). Specifically, the reference gene sets “c2.cp.all.v2022.1.Hs.symbols.gmt” were derived from the MSigDB database (https://www.gsea-msigdb.org/gsea/msigdb/collections.jsp, accessed on 8 July 2025). The pre-ranked gene list, ordered by the log_2_FC obtained from the differential expression analysis, was used as input for GSEA. Significantly enriched results were identified based on the cut-off values of *p*.adj < 0.05, false discovery rate (FDR) < 0.05, and |normalized enrichment score (NES)| > 1.5. The top six most significantly enriched pathways were visualized by the “ggplot2” package (version 3.4.4).

### 2.4. Gene Set Variation Analysis (GSVA)

To assess the enrichment of the gene sets at the individual sample level and uncover potential pathway activity changes associated with sarcopenia, GSVA [36] was performed using the “GSVA” package (version 1.50.0). Gene sets “c2.cp.v7.4.symbols.gmt” were sourced from the MSigDB database (accessed on 8 July 2025). Pathways that met the criteria of |t-scores| > 4 and *p*.adj < 0.05 were deemed significantly altered. The top 10 pathways with the most significant upward and downward adjustments were presented in a bar chart.

### 2.5. Weighted Gene Co-Expression Network Analysis (WGCNA)

In the training set, genes were filtered based on their median absolute deviation (MAD) values, with the top 25% exhibiting the smallest MAD being discarded. The “WGCNA” package [37] (version 1.73) was applied to construct a co-expression network. Pairwise correlation coefficients among all retained genes were calculated across samples. These correlations were transformed into connection strengths using a soft-thresholding power (β), generating a weighted adjacency matrix. The topological overlap measure (TOM) was derived from the adjacency matrix to quantify gene interconnectedness. Genes were then clustered hierarchically using TOM-based dissimilarity (1-TOM), and dynamic tree cutting (minModuleSize = 30, mergeCutHeight = 0.25) was applied to define distinct co-expression modules. Each module was assigned a unique color. Finally, key genes within biologically relevant modules were identified based on module membership (MM) > 0.8 and gene significance (GS) > 0.1.

### 2.6. Identification and Functional Analysis of SAR-CUP DEGs

Common genes among the DEGs, cuproptosis-related genes, and key WGCNA module genes were identified using the “VennDiagram” package [38] (version 1.7.3) and designated as sarcopenia-associated cuproptosis (SAR-CUP) DEGs. The expression levels of these candidate genes within the training set were evaluated using the Wilcoxon test to refine the selection and reduce the risk of high false discovery rates [39].

To elucidate the potential biological functions and pathways associated with the SAR-CUP DEGs, we performed Gene Ontology (GO) and Kyoto Encyclopedia of Genes and Genomes (KEGG) analyses via the “clusterProfiler” package (version 4.10.0). Significant terms were identified with a threshold of *p*.adj < 0.05.

### 2.7. Machine Learning for the Selection of the Hub Genes

To further identify the hub genes for diagnosing sarcopenia, three machine learning algorithms were employed: the least absolute shrinkage and selection operator (LASSO) regression, random forest (RF), and support vector machine (SVM). LASSO was conducted using the “glmnet” package [40] (version 4.1.10), for simultaneous regularization and feature selection, thereby improving both predictive accuracy and model interpretability. LASSO coefficient selection was performed using 10-fold cross-validation. RF was conducted via the “randomForest” package [41] (version 4.7.1.2), which addressed potential overfitting issues inherent in a single decision tree by aggregating predictions from multiple decision trees, thereby improving predictive reliability. SVM analysis was performed via the “e1071” package (version 1.7.16) using 10-fold cross-validation. It is a classical supervised learning method particularly well-suited for handling high-dimensional data and nonlinear classification problems. Potential diagnostic hub genes were identified based on the intersection of the results derived from these three algorithms. The chromosomal locations of the hub genes were visualized using the “circlize” package [42] (version 0.4.16).

### 2.8. Construction of a Regulatory Network

To investigate the regulatory mechanisms of the hub genes, we predicted their transcription factors (TFs) and micro-RNAs (miRNAs) using the NetworkAnalyst database [43] (https://www.networkanalyst.ca/, accessed on 10 July 2025). This comprehensive online platform facilitates visual transcriptome analytics and enables the construction of diverse biological networks, including protein–protein interaction (PPI) networks, gene regulatory networks, and co-expression networks. The resulting TF–miRNA–gene regulatory network was visualized using the Cytoscape software (version 3.10.2).

### 2.9. Construction of a Gene-Gene Interaction Network of the SAR-CUP DEGs

In order to further predict the potential functions of the SAR-CUP DEGs and construct a comprehensive gene interaction network, candidate genes that may functionally associate with the SAR-CUP DEGs were retrieved from the GeneMANIA database [44] (https://genemania.org/, accessed on 9 July 2025). This platform integrates diverse functional association data, such as physical and genetic interactions, pathways, co-expression, co-localization, and shared protein domains, to expand the network around query genes and offer biological insights.

### 2.10. Construction and Validation of a Diagnostic Model

To evaluate the diagnostic power of the hub genes for sarcopenia, we initially analyzed two hub genes individually, then integrated them into a combined diagnostic model, and finally validated the model using the independent dataset GSE136344. Briefly, receiver operating characteristic (ROC) analysis was performed via the “pROC” package [45] (version 1.18.0), and the results were visualized using the “ggplot2” package. The area under the curve (AUC) and its 95% confidence interval (CI) were calculated to quantify the diagnostic potential of these genes. An AUC value greater than 0.7 was considered indicative of good diagnostic capability [46]. To illustrate the discrepancy between predicted probabilities and actual probabilities and evaluate model fit, we plotted diagnostic calibration curves using “rms” package (version 6.4.0) and “ResourceSelection” package (version 0.3-5). The package “rmda” (version 1.6) was employed to conduct decision curve analysis (DCA), enabling the estimation of the clinical utility and net benefit. A nomogram model was constructed using the “rms” package (version 6.4.0) to provide a user-friendly, graphical tool for individualized prediction.

### 2.11. Cell Culture

C2C12 mouse myoblasts were purchased from the China Center for Type Culture Collection (CCTCC, Wuhan, China). Cells were cultured in Dulbecco’s Modified Eagle Medium (DMEM, Hyclone, SH30022.01, Logan, UT, USA) supplemented with 10% fetal bovine serum (FBS; Four Seasons Green, 11011-8611, Huzhou, China) and 1% penicillin-streptomycin (Gibco, 15140122, Grand Island, NY, USA), at 37 °C in a humidified atmosphere containing 5% CO_2_. For the construction of the sarcopenia cell model, the culture medium of the model group was replaced with fresh 10% FBS DMEM supplemented with 40 g/L D-galactose (Beyotime Biotechnology, ST1219-25g, Shanghai, China) at 70–80% cell confluence. The cells were subsequently cultured for an additional 48 h.

### 2.12. Reverse Transcription Quantitative Polymerase Chain Reaction (RT-qPCR)

Total cell RNA was extracted using an RNA extraction kit (TSP413, Tsingke Biotech, Beijing, China) and its purity and concentration were assessed via a microvolume spectrophotometry. cDNA was synthesized from RNA using the SynScript^®^Ⅲ RT SuperMix for qPCR kit (TSP314M, Tsingke Biotech, China). RT-qPCR was performed with the ArtiCanATM SYBR qPCR Mix kit (TSE501, Tsingke Biotech, China). GAPDH was employed as the reference gene. The expression data were analyzed using the 2^−ΔΔCT^ method. Primer sequences used for RT-qPCR are presented in Table 2.

### 2.13. Statistical Analysis

All statistical analyses associated with the bioinformatics analysis were performed using the R software (version 4.4.2). Differences between two groups were assessed with two-tailed *t*-tests. For comparisons across multiple groups, one-way ANOVA was applied, followed by Benjamini–Hochberg correction to control the false discovery rate. The difference in the continuous variables was assessed using the Wilcoxon test, whereas the correlation of the continuous variables was determined by the Pearson correlation. Statistical significance was defined as *p* < 0.05.

## 3. Results

### 3.1. Data Collection and Preprocessing

**Figure 1:**
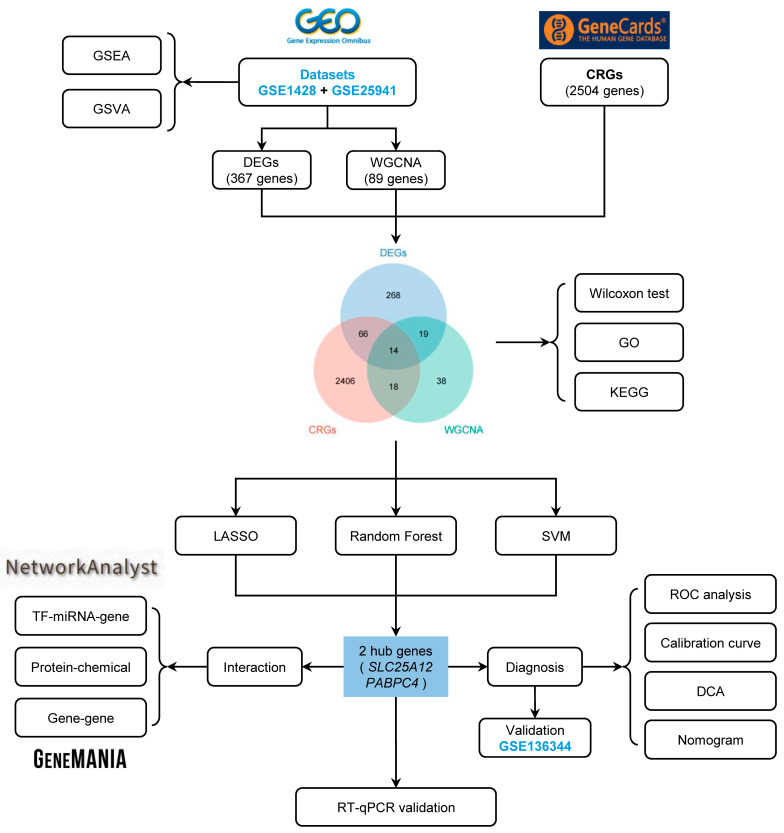
The flowchart of the overall study design.

**Figure 2:**
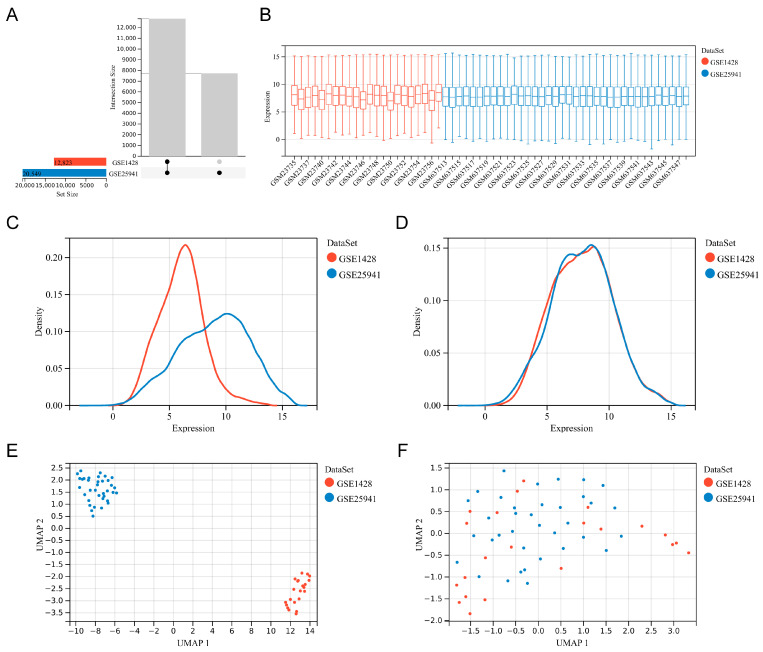
Data collection and preprocessing. (**A**) Intersection between the GSE1428 and GSE25941 datasets. (**B**) Box plot displaying normalized microarray data. (**C**,**D**) Density plots displaying expression differences before and after the batch effect removal. (**E**,**F**) UMAP plots displaying sample clustering between each dataset before and after the batch effect removal.

The overall study flowchart is illustrated in Figure 1. Microarray datasets GSE1428 and GSE25941, both associated with sarcopenia, were acquired from the GEO database (Figure 2A). After data cleaning and normalization, the two datasets were merged to enhance statistical power, followed by batch effect and outlier removal. Box plot demonstrated consistent expression distributions across datasets after correction, with aligned median values (Figure 2B). Primary density analysis further demonstrated robust internal dataset consistency following batch effect removal (Figure 2C,D). UMAP visualization revealed the integration of sample distributions, with initially separated clusters exhibiting extensive overlap after the merge (Figure 2E,F). These results suggested the efficacy of data preprocessing, laying the foundation for subsequent analyses.

### 3.2. Identification of the DEGs and Functional Analysis of the Training Set

**Figure 3:**
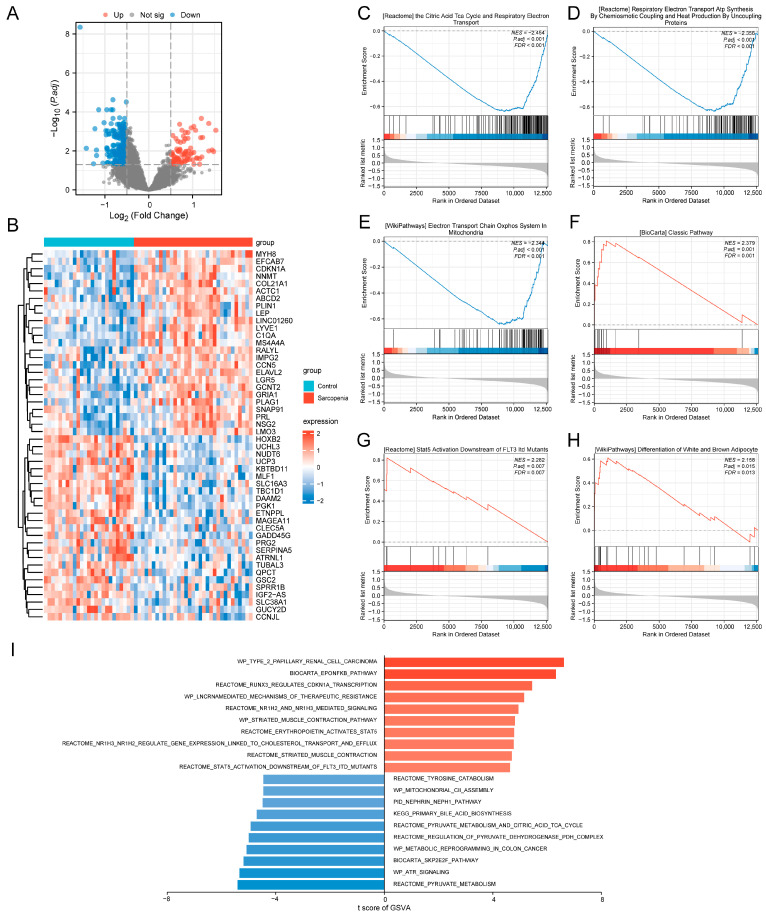
Identification of the DEGs and functional analysis of the training set. (**A**) Volcano plot of the DEGs. The red dots represent significantly up-regulated genes, and the blue dots represent significantly down-regulated genes. (**B**) Heatmap of the expression levels of the top 50 DEGs between the control and sarcopenia groups. (**C**–**H**) GSEA shows the pathways most relevant to the sarcopenia samples in the training set. (**I**) GSVA of the training set. The red columns indicate pathways that were significantly up-regulated in the sarcopenia group, and the blue columns indicate pathways that were significantly down-regulated in the sarcopenia group.

A total of 367 DEGs were identified, comprising 83 up-regulated and 284 down-regulated genes (Figure 3A). Expression patterns of the top 25 DEGs in each category are displayed in the heatmap (Figure 3B). To investigate sarcopenia-associated biological pathways, we performed GSEA on the training set. Based on the ranking of the NES, the top 3 negatively enriched pathways were: the citric acid cycle (TCA) and respiratory electron transport, respiratory electron transport ATP synthesis by chemiosmotic coupling and heat production by uncoupling proteins, and electron transport chain oxidative phosphorylation system in mitochondria (Figure 3C–E). The top 3 positively enriched pathways included: classical complement pathway, STAT5 activation downstream of FLT3 ITD mutants, and differentiation of white and brown adipocyte (Figure 3F–H). Additionally, GSVA was performed to quantify pathway activity dysregulation across samples. In the sarcopenia samples, pathways such as type 2 papillary renal cell carcinoma, EPO-NF-κb pathway, and RUNX3 regulates CDKN1A transcription were significantly enriched. Conversely, control samples exhibited enrichment of pyruvate metabolism, ATR signaling, and SKP2E2F pathway (Figure 3I). These results indicate that core pathological mechanisms underlying sarcopenia may involve mitochondrial dysfunction, chronic inflammation, and dysregulated adipose metabolism.

### 3.3. Identification of the Key Module Genes by WGCNA

**Figure 4:**
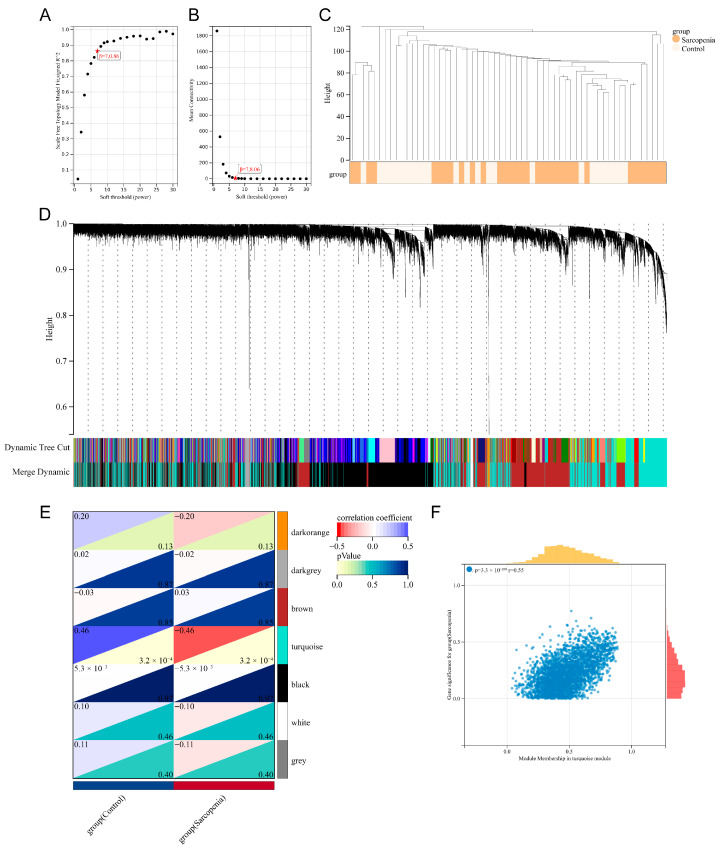
Identification of sarcopenia-related gene modules via WGCNA. (**A**,**B**) Selection of the best soft threshold (β = 7) based on the scale independence and mean connectivity. (**C**) Hierarchical clustering dendrogram of samples from control and sarcopenia groups. (**D**) Module division dendrogram. Different color represents different gene module. (**E**) Heatmap of the correlations between module eigengenes and sarcopenia. For each block, the upper triangle represents the correlation coefficient, while the lower triangle represents the *p*-value. (**F**) Scatter plot of the correlation between module membership and gene significance in turquoise gene module.

WGCNA was performed on the training set to identify biologically meaningful gene modules. As illustrated in Figure 4A,B, a soft threshold of β = 7 was chosen to construct a network based on the scale-free topology model fit (R^2^ = 0.86) and mean connectivity. The clustering pattern of gene expression profiles between the control and sarcopenia groups is displayed in Figure 4C. Hierarchical clustering with dynamic tree cutting identified 7 distinct co-expression modules, each labeled with a different color (Figure 4D). The gray module, which contained genes not assigned to any cluster, was excluded from subsequent analyses. Correlation analysis revealed that the turquoise module exhibited the strongest association with sarcopenia (r = −0.46, *p* < 0.05) (Figure 4E). Within this module, module membership and gene significance exhibited a significant positive correlation (r = 0.55, *p* < 0.05) (Figure 4F). Consequently, 89 genes in the turquoise module were identified as sarcopenia-related candidates.

### 3.4. Selection and Functional Analysis of the SAR-CUP DEGs

**Figure 5:**
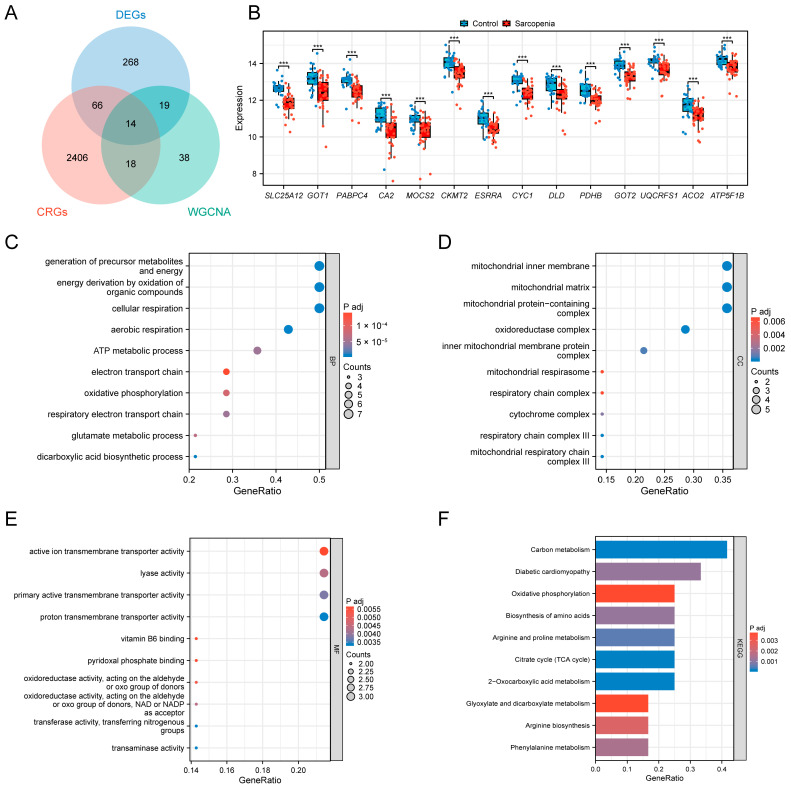
Identification and functional enrichment analysis of the SAR-CUP DEGs. (**A**) Venn diagram illustrating the intersection among DEGs, sarcopenia-related genes, and cuproptosis-related genes. (**B**) Box plot of expression of the 14 SAR-CUP DEGs in the training set. (**C**–**E**) GO enrichment results of the 14 SAR-CUP DEGs in biological process (**C**), cellular component (**D**), and molecular function (**E**) terms. (**F**) KEGG enrichment results of the 14 SAR-CUP DEGs. *** *p* < 0.001.

A total of 14 SAR-CUP DEGs were identified by intersecting 367 DEGs, 89 sarcopenia-related genes, and 2504 cuproptosis-related genes (Figure 5A). These key genes included *SLC25A12*, *GOT1*, *PABPC4*, *CA2*, *MOCS2*, *CKMT2*, *ESRRA*, *CYC1*, *DLD*, *PDHB*, *GOT2*, *UQCRFS1*, *ACO2*, and *ATP5F1B*. All of the SAR-CUP DEGs exhibited significantly differences between the control and sarcopenia groups (*p* < 0.05) (Figure 5B). Functional enrichment analyses were then performed to characterize their biological roles. GO terms, namely biological process (BP), cellular component (CC), and molecular function (MF), as well as KEGG pathways with *p*.adj < 0.05 were considered significantly enriched. The top 10 enriched terms in each category were visualized. The most enriched BP terms included generation of precursor metabolites and energy, energy derivation by oxidation of organic compounds, and cellular respiration (Figure 5C). Key CC terms comprised mitochondrial inner membrane, mitochondrial matrix, and mitochondrial protein-containing complex (Figure 5D). In MF, significant enrichment was observed for active ion transmembrane transporter activity, lyase activity, and primary active transmembrane transporter activity (Figure 5E). KEGG pathway analysis further highlighted significantly enrichment in metabolic pathways, carbon metabolism, and 2-Oxocarboxylic acid metabolism (Figure 5F).

### 3.5. Identification of the Hub Genes by Machine Learning

**Figure 6:**
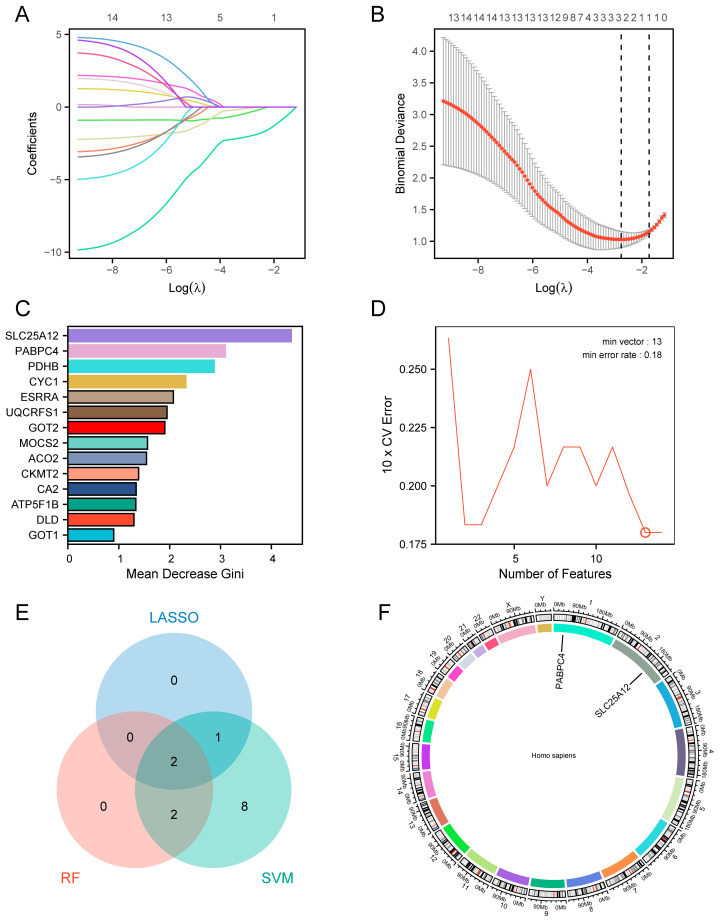
Identification of the hub genes by machine learning. (**A**) LASSO regression coefficient path plot. Each line represents the regression coefficient for a variable. (**B**) LASSO regression cross-validation curve. Each red dot represents the average binomial deviance calculated via cross-validation at the corresponding Log(λ) value. The left dashed line represents the optimal lambda value for the evaluation metric (lambda.min). The right dashed line represents the lambda value (lambda.1SE) of the model within one standard error of the optimal value for the evaluation metric. (**C**) RF feature importance bar plot. (**D**) Cross-validation error curve for SVM feature selection. The point marked with a red circle indicates the minimum number of feature variables corresponding to the lowest error rate in the cross-validation results. (**E**) Venn diagram illustrating the overlap among the three machine learning outcomes. (**F**) Chromosome localization map of the hub genes. Each color represents a pair of chromosomes.

Three machine learning algorithms were applied to select the potential hub genes for sarcopenia diagnosis. LASSO regression analysis identified 3 crucial genes (lambda.min = 0.063) (Figure 6A,B). Using the Mean Decrease Gini index, the RF algorithm ranked SAR-CUP DEGs and selected 4 candidate genes (Figure 6C). In the SVM analysis, the cross-validation curve revealed that the error rate minimized at 0.18 with 13 features, indicating this optimal feature set corresponded to 13 genes (Figure 6D). Integration of the results from all 3 algorithms identified 2 hub genes (Figure 6E), namely solute carrier family 25 member 12 (*SLC25A12*) and poly(A) binding protein cytoplasmic 4 (*PABPC4*). The chromosomal locations of the common genes are visualized in Figure 6F. *SLC25A12* and *PABPC4* are located on chromosomes 2 and 1, respectively.

### 3.6. Construction of the TF-miRNA-Gene, Protein-Chemical, and Gene-Gene Interaction Network

**Figure 7:**
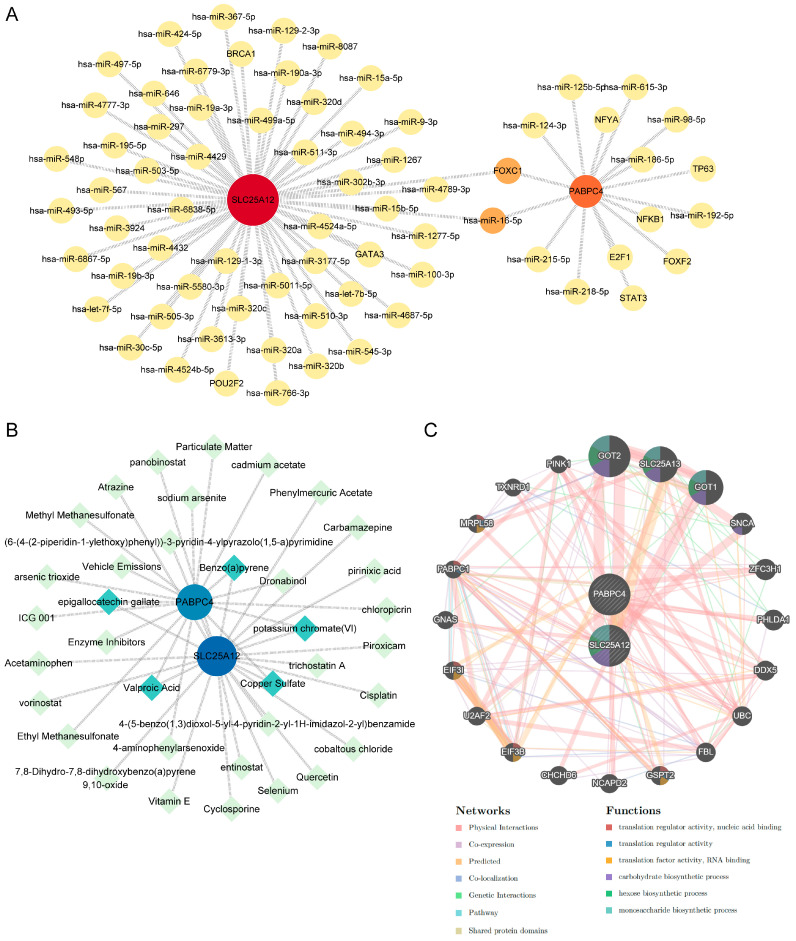
Regulatory networks of the hub genes. (**A**) TF-miRNA-gene regulatory network. (**B**) Protein-chemical interaction network. (**C**) Gene-gene interaction network.

To further elucidate the regulatory network of these hub genes and identify potential diagnostic and therapeutic targets, we predicted the TFs, miRNAs, and chemicals associated with them using the NetworkAnalyst database. The TF-miRNA-gene network consisted of 73 nodes and 73 edges (Figure 7A). A total of 10 TFs and 61 miRNAs were predicted, among which forkhead box transcription factor C1 (FOXC1) and has-miR-16-5p were associated with both hub genes. Furthermore, the protein-chemical interaction network identified 36 chemicals potentially interacting with *SLC25A12* and *PABPC4* (Figure 7B). Among these, five chemicals were linked to both genes, including benzo(a)pyrene, copper sulfate, epigallocatechin gallate, potassium chromate (VI), and valproic acid. Additionally, we constructed gene-gene interaction network to elucidate gene functions and discover genes with synergistic interactions (Figure 7C). Strong physical interactions were demonstrated between the two hub genes and the 20 predicted associated genes. In addition, *SLC25A12*, *GOT2*, *SLC25A13*, and *GOT1* were all involved in carbohydrate, hexose, and monosaccharide biosynthetic process, which indicated their importance in cellular metabolism.

### 3.7. Construction of the Diagnostic Model

**Figure 8:**
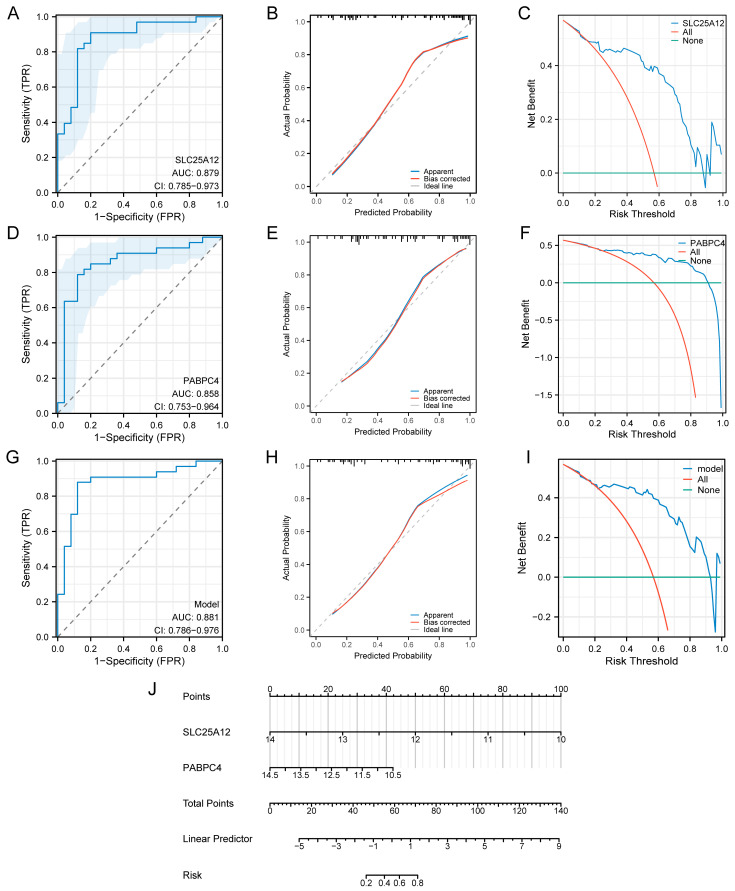
Diagnostic model of *SLC25A12* and *PABPC4* in the training set. (**A**) ROC analysis of *SLC25A12* for diagnostic efficacy. The dashed diagonal represents the performance of a classifier with no discriminative ability (i.e., random guessing). The blue shadow area represents the confidence interval. (**B**) Calibration curve of *SLC25A12* to assess the agreement between predicted and actual probabilities. (**C**) DCA plot of *SLC25A12* to evaluate net benefit across different risk thresholds. (**D**) ROC analysis of *PABPC4*. (**E**) Calibration curve of *PABPC4*. (**F**) DCA plot of *PABPC4*. (**G**) ROC analysis of the combined diagnostic model. (**H**) Calibration curve of the combined model. (**I**) DCA plot of the combined model. (**J**) Nomogram constructed based on *SLC25A12* and *PABPC4*.

To evaluate the diagnostic and predictive performance of the hub genes, we conducted ROC analysis, calibration curve assessment, DCA, and nomogram analysis. For *SLC25A12*, the ROC curve exhibited an AUC of 0.879 (95% CI = 0.785–0.973), indicating excellent diagnostic efficacy (Figure 8A). The calibration curve showed favorable agreement between predicted and actual probabilities (Figure 8B). DCA plot revealed that *SLC25A12* generated a positive net benefit across a wide range of risk thresholds (Figure 8C). For *PABPC4*, the ROC curve yielded an AUC of 0.858 (95% CI = 0.753–0.964), suggesting notable diagnostic ability (Figure 8D). Its calibration curve also demonstrated reasonable consistency between predicted and actual probabilities (Figure 8E). DCA plot indicated that *PABPC4* provided a positive net benefit within specific risk thresholds (Figure 8F).

When *SLC25A12* and *PABPC4* were integrated into a combined model, the ROC curve achieved a higher AUC of 0.881 (95% CI = 0.786–0.976), reflecting enhanced diagnostic performance (Figure 8G). The calibration curve confirmed the model’s reliability in probability prediction (Figure 8H). DCA plot also showed that the combined model offered an excellent net benefit across most risk thresholds (Figure 8I). Furthermore, a nomogram was developed based on *SLC25A12* and *PABPC4*, which quantified risk scores by integrating points assigned to each gene (Figure 8J). These analyses highlighted *SLC25A12* and *PABPC4* as potential crucial diagnostic biomarkers for sarcopenia.

### 3.8. Validation of the Diagnostic Model

**Figure 9:**
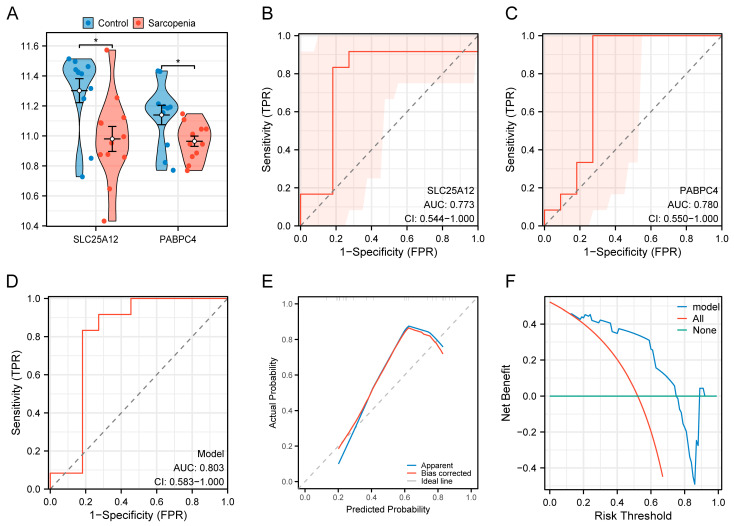
Validation of the diagnostic model in the GSE136344 dataset. (**A**) Expression levels of *SLC25A12* and *PABPC4* in the validation set. (**B**) ROC curve of *SLC25A12*. The dashed diagonal represents the performance of a classifier with no discriminative ability (i.e., random guessing). The red shadow area represents the confidence interval. (**C**) ROC curve of *PABPC4*. (**D**) ROC curve of the combined model. (**E**) Calibration curve of the combined model. (**F**) DCA plot of the combined model. * *p* < 0.05.

To evaluate the robustness of the diagnostic model, validation was performed using the GSE136344 dataset. The expression levels of *SLC25A12* and *PABPC4* were significantly downregulated in the validation cohort, consistent with the previous findings (Figure 9A). The ROC analyses for *SLC25A12* and *PABPC4* showed AUC values of 0.733 (95% CI = 0.544–1.000) and 0.780 (95% CI = 0.550–1.000), respectively (Figure 9B,C). Moreover, the combined diagnostic model yielded an AUC of 0.803 (95% CI = 0.583–1.000) (Figure 9D). The calibration curve showed that the predicted curve deviate above the ideal line over a portion of the range, suggesting a tendency to underestimate diagnostic risk (Figure 9E). DCA plot further indicated a favorable net benefit (Figure 9F). Although the AUC values for both individual genes and the integrated model in the validation set were lower than those in the training set, these AUC values still exceeded 0.7, falling within an acceptable range. Collectively, these results support the diagnostic model as a promising tool with potential clinical applicability.

### 3.9. Preliminary in Vitro Validation of the Hub Genes

**Figure 10:**
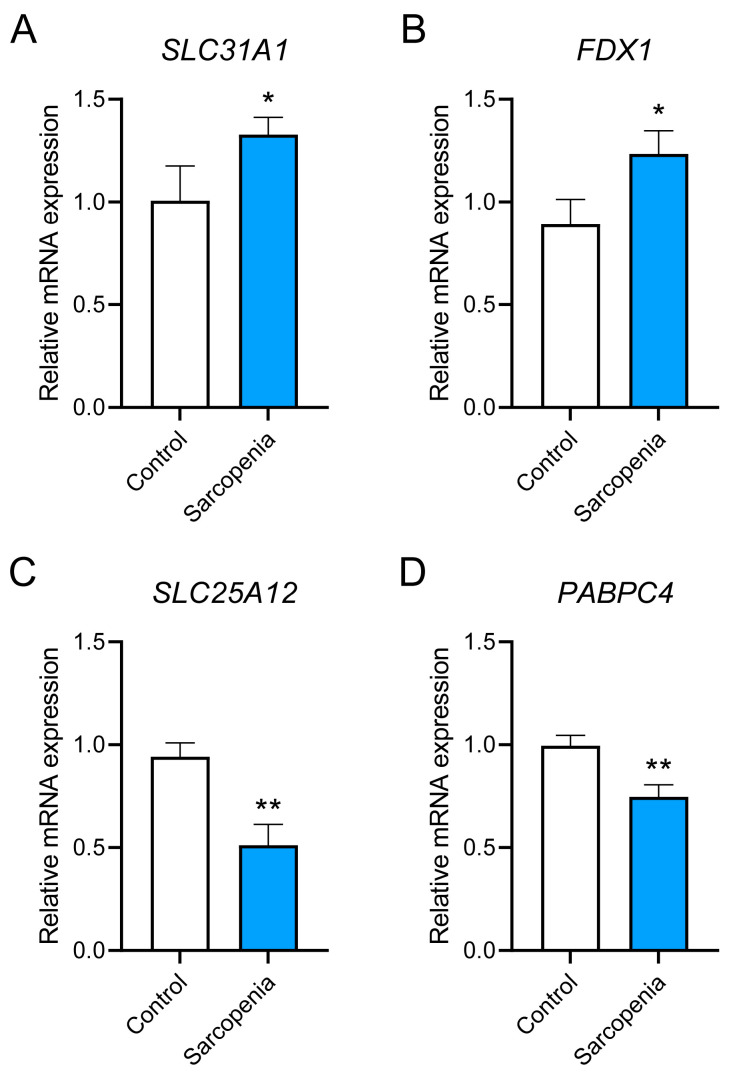
Validation of the mRNA expression levels of the hub genes. (**A**,**B**) The mRNA expression levels of the cuproptosis marker genes *SLC31A1* and *FDX1*. (**C**,**D**) The mRNA expression levels of the diagnostic hub genes *SLC25A12* and *PABPC4*. * *p* < 0.05, ** *p* < 0.01.

The expression levels of the two hub genes were measured in a D-gal-induced sarcopenia cell model. As shown in Figure 10, cuproptosis marker genes *SLC31A1* [47] and *FDX1* [48] were significantly up-regulated (*p* < 0.05), while the expression levels of *SLC25A12* and *PABPC4* were significantly down-regulated (*p* < 0.01). This indicated that cuproptosis may occur in sarcopenic cell models and the hub genes may serve as a crucial link between cuproptosis and sarcopenia.

## 4. Discussion

With the rapid increase in the aging population, sarcopenia, a disease highly prevalent among the elderly, has drawn growing attention from both researchers and clinicians [49]. Sarcopenia not only means a decline in skeletal muscle mass and strength, but can also trigger a series of reactions that pose multiple threats to overall health [50]. Copper is an essential trace element that plays a vital role in human health and disease. Copper ions participate in a wide range of physiological functions, including energy metabolism, antioxidant activity, neurotransmitter synthesis, and iron regulation [51]. Maintaining copper homeostasis facilitates the normal physiological functions of multiple tissues and organs [52]. In the human body, more than half of the copper is stored in bone and muscle, where it can exert complex and integral influences on muscle structure and function [53,54]. During aging, copper ions have a tendency of accumulating within skeletal muscle cells, ultimately contributing to muscle atrophy by promoting various forms of cell death, including apoptosis, pyroptosis, ferroptosis, and cuproptosis [23,55,56]. Although studies have revealed the involvement of cuproptosis in diverse diseases, including neurodegenerative disorders and cardiovascular diseases [53], its potential role in sarcopenia has not yet been fully explored. Given that skeletal muscle is one of the most metabolically active organs with a strong dependence on copper [14], we hypothesize that cuproptosis may serve as a key contributor to the progression of sarcopenia.

In this study, we integrated bioinformatics analyses, machine learning algorithms, and in vitro validation to investigate the association between cuproptosis and sarcopenia. Among the 367 identified DEGs, the majority were downregulated. GSEA and GSVA indicated that the pathogenesis of sarcopenia involved multiple pathways, including impaired mitochondrial energy metabolism, chronic inflammatory activation, abnormal adipocyte differentiation, and dysregulation of cell regeneration and cycle regulation. By integrating DEGs, cuproptosis-related genes, and WGCNA results, we were able to identify 14 SAR-CUP DEGs, which exhibited strong associations with mitochondrial structure and function, particularly in oxidative phosphorylation and the TCA cycle. Three machine learning algorithms further screened out two critical predictive genes: *SLC25A12* and *PABPC4*. Subsequent analyses characterized their regulatory networks and diagnostic value, followed by preliminary validation through in vitro experiments. These findings may offer a solid framework for comprehending the molecular mechanisms of sarcopenia and advancing diagnostic and therapeutic strategies.

*SLC25A12* encodes the mitochondrial aspartate-glutamate carrier 1 (AGC1), which mediates the export of aspartate from the mitochondrial matrix to the cytoplasm while simultaneously countertransporting glutamate and protons into the mitochondria. This process is crucial for maintaining cellular energy metabolism and neurotransmitter synthesis [57,58]. *SLC25A12* is highly expressed in the brain, heart, and skeletal muscle [59]. Previous studies indicate that this gene has been closely associated with neurological disorders. For example, several single nucleotide polymorphisms (SNPs) in *SLC25A12* have been linked to autism spectrum conditions (ASC) [60,61]. However, the association between *SLC25A12* and ASC remains controversial [62]. Emerging findings also highlight its importance in skeletal muscle physiology. Specifically, downregulation of *SLC25A12* in mouse skeletal muscle impairs mitochondrial NADH shuttle activity, leading to a significant reduction in ATP production [63]. Furthermore, AGC1 deficiency syndrome caused by *SLC25A12* mutation may present with generalized hypotonia in addition to central nervous system symptoms [64]. Evidence from Dutch shepherd dogs further illustrates that mutations in *SLC25A12* can trigger an imbalance in muscle redox status, creating a highly oxidized pro-inflammatory environment. This leads to substantial accumulation of inflammatory markers and oxidative stress metabolites within the muscle, directly impairing myocyte function and ultimately manifesting as inflammatory myopathy [65]. Additionally, downregulation of *SLC25A12* expression has been associated with age-related muscle dysfunction [66], indicating its potential role in sarcopenia. Collectively, these findings suggest that *SLC25A12* dysfunction compromises mitochondrial energy metabolism, redox balance, and muscle integrity, thereby providing a potential mechanistic link between altered AGC1 activity and muscle weakness characteristic of sarcopenia.

*PABPC4* belongs to the cytoplasmic polyadenylate-binding protein (PABP) family and contains a characteristic RNA-binding domain (RBD). By binding specifically to the 3′-polyadenylate tail of mRNA, it regulates mRNA stability and translation efficiency [67,68]. Functionally, *PABPC4* is crucial for the expression of erythroid mRNAs, erythroid differentiation, and cell growth and metabolism [69,70]. It also closely related to cancers such as triple-negative breast cancer, liver cancer, and renal cell carcinoma [71,72,73]. Importantly, *PABPC4* is highly enriched in skeletal muscle, where it participates in mitochondrial regulation under metabolic stress. Decreased expression of *PABPC4* enhances the ubiquitination and degradation of the nuclear receptor corepressor 1 (NCoR1), leading to increased activity of metabolism-related genes such as PPARγ. This subsequently promotes mitochondrial biogenesis and oxidative metabolism, thereby improving the metabolic adaptability of skeletal muscle [74]. Although direct evidence linking *PABPC4* to structural or functional regulation of skeletal muscle cells is lacking, its role in mitochondrial homeostasis, energy metabolism, and stress responses suggests a potential relevance to sarcopenia.

To gain a more comprehensive understanding of these two hub genes, we predicted the TFs and miRNAs associated with hub genes at both the transcriptional and post-transcriptional levels. Among the predicted regulators, the transcription factor FOXC1 and the microRNA has-miR-16-5p were both closely associated with the two hub genes. FOXC1, a member of the forkhead box (FOX) family, is essential for embryonic development and the maintenance of adult stem and progenitor cell pools [75]. In skeletal muscle, it has been identified as a potential transactivation target of mutant glucocorticoid receptors [76] and can suppress osteoblast differentiation by inhibiting the BMP-SMAD signaling pathway [77]. It is also considered one of the key TFs regulating genes crucial for sarcopenia [78]. Meanwhile, miR-16-5p can directly targets SESN1, suppressing its expression to activate the p53 signaling pathway, thereby inhibiting cell proliferation and promoting apoptosis [79]. Plus, miR-16-5p levels are significantly elevated in circulating exosomes derived from sarcopenia patients, which suggests its potential as a diagnostic biomarker for sarcopenia [80]. We also predicted genes that may interact with the two hub genes, with *GOT2*, *SLC25A13*, and *GOT1* showing particularly strong associations. Notably, *GOT2* and *GOT1* were also present among the 14 previously identified SAR-CUP DEGs, further confirming their relevance. These enzymes catalyze the transamination reaction between glutamate and oxaloacetic acid, yielding α-ketoglutarate and aspartate. This reaction links carbohydrate metabolism to the TCA cycle, supplying energy to skeletal muscle [81]. Additionally, the protein-chemical interaction network revealed 5 strongly correlated compounds. Among them, benzo(a)pyrene and potassium chromate (VI) are both group 1 carcinogens [82]. In cancer cachexia models, valproic acid can act as a histone deacetylase inhibitor that inhibits C/EBPβ activity, thereby reducing muscle protein breakdown and mitigating muscle cell atrophy [83]. Epigallocatechin gallate (EGCG) is a natural polyphenolic compound primarily found in green tea, exhibiting multiple effects including antioxidant and anti-inflammatory properties [84]. Interestingly, EGCG displays a dual role in cuproptosis: it promotes copper accumulation and cell death in cancer cells while protecting normal cells from oxidative stress through metal chelation [85,86]. Taken together, these findings suggest that transcriptional and post-transcriptional regulators, metabolic enzymes, and environmental factors converge on pathways related to mitochondrial function, oxidative stress, and energy metabolism.

However, this study still has several limitations. First, the sample size is limited, which may affect the generalizability of the results. Furthermore, the experimental validation primarily relies on a single cell line, lacking verification across different cell lines, in vivo experiments, and clinical trials. Additionally, the causal chain between cuproptosis and sarcopenia remains incompletely understood. Finally, the connections between the identified hub genes and other established sarcopenia biomarkers have not been fully elucidated, necessitating further mechanistic investigation. In future studies, we will strive to design rigorous experiments to explore the mechanisms underlying cuproptosis and sarcopenia, with the aim of developing optimal intervention strategies.

## 5. Conclusions

This study systematically explored the potential connection between cuproptosis and sarcopenia. Our findings suggest that mitochondrial dysfunction, impaired energy metabolism and oxidative stress may represent shared pathological mechanisms underlying both conditions, with *SLC25A12* and *PABPC4* emerging as key molecular mediators. These insights highlight that strategies combining copper monitoring and targeted interventions with rational pharmacological and nutritional modulation may provide novel approaches for the prevention and treatment of sarcopenia.

## Figures and Tables

**Table 1:** Detailed information of the selected GEO datasets.

Dataset	Control	Sarcopenia	Platform	Species	Tissue	Type
GSE1428	10	12	GPL96	Homo sapiens	vastus lateralis muscle	Expression profiling by array
GSE25941	15	21	GPL570	Homo sapiens	vastus lateralis muscle	Expression profiling by array
GSE136344	11	12	GPL5175	Homo sapiens	vastus lateralis muscle	Expression profiling by array

**Table 2:** Primer sequences used for RT-qPCR.

Gene	Sequence (5′-3′)	Size (bp)
*GAPDH*	F: TGTTTCCTCGTCCCGTAGA R: GATGGCAACAATCTCCACTTTG	116
*SLC31A1*	F: AACCACACGGACGACAACAT R: CAGACCCTCTCGGGCTATCT	246
*FDX1*	F: CGCTAACGACCAAGGGGAAA R: GTAGAGCAAGCCAACGTTCC	109
*SLC25A12*	F: GCGGAAATCCTTGCTGGAGGTT R: TGACTCTCGGTCCTGTGGTGAT	118
*PABPC4*	F: GACCAAAGCTGTCACCGAGA R: GGCACAAAATAGCCACCAGC	195

## Data Availability

The datasets used for the study are publicly available in the GEO database (https://www.ncbi.nlm.nih.gov/geo/, accessed on 8 July 2025), GeneCards database (https://www.genecards.org/, accessed on 8 July 2025), MSigDB database (https: //www.gsea-msigdb.org/gsea/msigdb/index.jsp, accessed on 8 July 2025), GeneMANIA database (https://genemania.org/, accessed on 9 July 2025), NetworkAnalyst database (https://www.networkanalyst.ca/, accessed on 10 July 2025).
